# Characteristics and Influencing Factors of Real-Life Violence Exposure Among Chinese College Students

**DOI:** 10.3389/fpsyg.2020.01312

**Published:** 2020-06-26

**Authors:** Weiling Wang, Yuping Wang, Yuyan Qian, Yuanfang Yu

**Affiliations:** ^1^School of Humanities and Social Sciences, Institute of Social Psychology, Xi’an Jiaotong University, Xi’an, China; ^2^Department of Sociology, School of Humanities and Social Sciences, Xi’an Jiaotong University, Xi’an, China

**Keywords:** characteristics, influential factors, violence exposure, family violence, community violence, college students

## Abstract

This study aimed to explore the characteristics and influencing factors of violence exposure in real life among Chinese college students. A sample of 375 college students was randomly selected to complete three questionnaires. The results indicated that participants had higher scores as victims and witnesses on violence exposure in community than they did in family. Male students had higher scores than females in both family and community violence exposure. Subjects with lower father’s education level scored significantly higher than others in family violence exposure by victimization and community violence exposure by witnessing and victimization. Participants growing up in rural areas had significantly higher scores than others in family violence exposure by victimization and community violence exposure by witnessing. Finally, those subjects with siblings reported higher scores than those from only child families in family violence exposure by witnessing. Multiple regression analysis showed that deviant behaviors of peers, gender, and single-child status were significant influencing factors of respondent violence exposure. More efforts should be taken to effectively cope with existing violence exposure in college students and minimize the potential of future exposure.

## Introduction

Violence refers to “the intentional use of physical force or power, threatened or actual, against oneself, another person, or a group or community, which either results in or has a high likelihood of resulting in injury, death, psychological harm, mal-development, or deprivation” and was officially announced as a crucial risk factor for public health (World Health Organization, 1996). More than 1.3 million people worldwide die from various types of violence each year, accounting for about 2.5% of total mortality (World Health Organization et al., 2014). In addition to causing death, violence can cause physical problems, such as injury, disability, depression, drug abuse, and high-risk sexual behavior (World Health Organization, 2015), resulting in heavy burdens on the health, welfare, and justice sectors.

Children and adolescents who have been victims of violence are a major societal concern. Studies revealed that children and adolescents suffer higher rates of exposure to violence than adults (Hashima and Finkelhor, 1999; Finkelhor, 2008). About half of high school youth reported being threatened, slapped, hit, or punched in the home, school, or neighborhood, and up to a third of high school youth indicated being beaten or mugged in the school or neighborhood, attacked with a knife or stabbed, or shot at by another person (Singer et al., 1995). Marcus and Reio (2002) reported that physical altercations resulting from violence among college students reached a rate of 63%, of which 9.1% suffered injuries and required medical care. The situation seems more serious in China. According to one study, the prevalence of only campus violence among college students in Guangzhou city was 69.9% (Wang et al., 2012), calling public attention about college student violence exposure in China.

Exposure to violence also has strong negative impacts on children and adolescents. High exposure in daily life explains a large amount of behavioral and mental problems (Fergusson et al., 2008; Gilbert et al., 2009). Prospective longitudinal studies consistently showed that maltreated children have lower educational achievement than their peers and are more likely to receive special education (Jonson-Reid et al., 2004; Boden et al., 2007). Adolescent violence exposure also increased the risk of behavior problems including intrinsic (anxiety, depression, anger) and extrinsic (aggression, acting out) behavior, post-traumatic stress disorder, and even the likelihood of illegal and criminal activities (Singer et al., 1995; Banyard et al., 2001; Manly et al., 2001; Whiffen and Macintosh, 2005; Herrenkohl and Herrenkohl, 2007; Fergusson et al., 2008). Furthermore, violence in the living environment can lead to suicidal behavior of college students with irreversible consequences (Chen, 2007).

The entire society suffers from the high occurrence of violence and its adverse impacts on children and adolescents. Exposure to violence can increase the risk of injuries, infectious diseases, such as reproductive health problems and AIDS, and non-communicable diseases, such as heart disease and cancer (Meyers et al., 2018). Apart from the huge increases in medical expenses, national and local economies are indirectly affected by eroded human capital. Social inequality and discrimination would also be exacerbated, further hindering social development and increasing uncertainty (World Health Organization, 2008). The cost of violence is enormous, and effective measures to prevent violence are essential and urgent to ensure long-term societal stability.

Unfortunately, there are several limitations of existing studies. Firstly, most researches on violence exposure focused on children and adolescents but overlooked the population of college students aged at 18–22 (Geiger and Castellino, 2011). Previous studies have shown that college students experience more violence exposure than other age groups (Singer et al., 1995; Chen, 2007; Finkelhor et al., 2009), underscoring the need for investigations of this population. Secondly, although studies suggested that direct violent victimization should have significant negative impacts on development in youth, the potential negative influences of witnessing violence should not be dismissed. It is urgent and crucial to conduct more studies to identify factors associated with both victimization and witnessing violence among college students. Thirdly, due to the influence of traditional Chinese culture, people may display higher acceptance and tolerance for violence. For example, unlike Western countries, parents and teachers in China usually consider beating children as a normal and acceptable way to educate them. Adolescents in China seem much more likely to be exposed to violence. Additionally, most Chinese adolescents experience the transition stage to adulthood during their college years when they first leave family and face the world by themselves, which is an essential period for the development of psychological maturity. Away from their parents, college students have more opportunities to be exposed to violence in their daily life as they are eager to explore and have more access to information, which can have negative effects on their physical and mental health.

Most domestic research on violence has been qualitative, mainly to investigate the incidence, type, and risk factors of violence. This study applies quantitative research methods and adopts international definitions and measurement tools to systematically and objectively study the characteristics and influencing factors of violence exposure among Chinese college students. With the approach, the results can be easily compared with other existing and future studies at home and abroad.

In this quantitative study, we aimed (1) to investigate the victimization and witnessed violence exposure in both the family and community and (2) to explore the influences of sociodemographic variables and peer factors on violence exposure in Chinese college students. We assumed that growing up in a rural area, lower parental education level, having siblings, and having peers with more deviant behaviors were risk factors for violent exposure among college students.

## Materials and Methods

### Procedure

A total of 410 college students were selected from Xi’an Jiaotong University, Shaanxi Normal University, Northwestern Polytechnic University, and Northwest University, which are “211” universities ranking in the top 100 in China and located in Xi’an, Shaanxi Province. The Research Ethics Committee of Xi’an Jiaotong University approved this study protocol. After being fully informed of the study purpose, 385 college students agreed to complete the surveys and sign written consent letters. Before the start of the research, researchers explained the definition of community and domestic violence to all participants. Community violence is interpersonal violence between individuals and people who are not closely related in the community in various formats including sexual assault, burglary, youth gangs, drug abuse, and ethnic and racial segregation (Krug et al., 2002). Domestic violence refers to physical, mental, and other violations committed by family members by means of beating, restricting personal freedom, and frequent insulting and threatening (United Nations, 2017). We used a convenience sampling method for this research project. During a mental health class, under the supervision of a researcher and a teacher, 385 participants anonymously finished the general questionnaire, Community Experience Questionnaire (CEQ), and Peer Group Questionnaire (PGQ) in about 30 min. Ten participants did not finish at least one item of the surveys, leaving 375 college students as the effective sample. The participation rate of this survey was 93.90%, and the effective response rate was 97.40%.

### Participants

A total of 375 college students completely finished the surveys. The mean age of the subjects was 20.03 years (*SD* = 1.410, range = 15–24). More than half (59.2%) of the subjects were male. The mean years of their parents’ education were 12.88 and 12.09 years for the father and mother, respectively (corresponding *SDs* of 3.41 and 3.07). Most subjects (94.7%) came from a two-parent family and others from a single-parent family. More than half (53.3%) lived in a nuclear family (family members include only parents and children), and others lived in a non-nuclear family (above family members plus grandparents or others). Around three-fifths of the subjects (58.1%) grew up in urban areas, and the others in rural areas. More than half (56.8%) of participants were singletons (only child), and others were non-singletons (one or more siblings). The percentages for Grade 1, Grade 2, and Grade 3 students were 40.3, 26.4, and 33.3%, respectively. Table 1 presents more details about the sample.

**TABLE 1:** Sociodemographic characteristics of participating college students.

**Characteristics**		***n* (375)**	**%**
Age	≤17	11	2.9
	18	44	11.7
	19	78	20.8
	20	97	25.9
	21	88	23.5
	22	48	12.8
	≥23	9	2.4
Gender	Male	222	59.2
	Female	153	40.8
Family structure	Single-parent family	20	5.3
	Two-parent family	355	94.7
Father’s years of	≤9	118	31.5
schooling	10–12	112	29.9
	13–16	119	31.7
	≥17	26	6.9
Mother’s years of	≤9	148	39.5
schooling	10–12	117	31.2
	13–16	101	26.9
	≥17	9	2.4
Family type	Nuclear family	200	53.3
	Non-nuclear family	175	46.7
Place of birth	Urban	218	58.1
	Rural	157	41.9
Single-child status	Only child	213	56.8
	Not only child	162	43.2
Grade	Grade 1	151	40.3
	Grade 2	99	26.4
	Grade 3	125	33.3

### Measures

#### General Questionnaire

The general questionnaire asked about sociodemographic information including age, gender, place of growing up, family members they lived with, parental education level, siblings, and family history of mental illness.

#### Community Experience Questionnaire (CEQ)

The CEQ (Schwartz and Proctor, 2000) was used to test the violence exposure of students in their real-life family and community environments. It was developed by Schwartz and Proctor in 2000 on the basis of the Community Violence Questionnaire designed by Richters and Saltzman (1990). The CEQ contains 26 items with 12 and 14 items evaluating violence exposure by direct victimization and witnessing, respectively. Each item had four questions. The first two were related to a certain family violence exposure and the exact time of its occurrence, and the latter two were associated with a certain community violence exposure and its time of occurrence. There were four subscales: family violence exposure by victimization (FVEV), community violence exposure by victimization (CVEV), family violence exposure by witnessing (FVEW), and community violence exposure by witnessing (CVEW). Each item referred to a certain type of violence exposure and scored from 0 (never) to 3 (many times). The subscale result was calculated by summing up all the scores of the questions in the subscale. The possible total scores of FVEV and CVEV ranged from 0 to 36, and those of FVEW and CVEW ranged from 0 to 42. The higher the total score, the higher the violence exposure (Schwartz and Proctor, 2000). In the current study, Cronbach’s alpha values for the four subscales (FVEV, FVEW, CVEV, CVEW) were 0.69, 0.77, 0.75, and 0.85 respectively, while that for the whole scale was 0.89.

#### Peer Group Questionnaire (PGQ)

We used the PGQ designed by Metzler et al. (1994) to assess the deviant behaviors of peers of college students. The PGQ consists of 13 items (e.g., “how many of your friends vandalize property?” and “how many of your friends get into fights?”) rated from 0 (none) to 4 (almost all of them). The total scores of deviant behaviors were determined by summing up the ratings they indicated in the questionnaire. Higher scores on an item implied more deviant behaviors by their peers (Metzler et al., 1994). The internal consistency reliability of the PGQ in this study was 0.729.

### Statistical Analysis

Descriptive analyses, paired sample *t*-tests, independent sample *t*-tests, analyses of variance (ANOVAs), and correlation analyses were performed in this study using SPSS software (IBM SPSS Statistics Version 23.0, Armonk, NY, United States). Paired sample *t*-tests were conducted to evaluate differences in violence exposure between family and community. Independent sample *t-*tests were used to assess differences in family violence exposure by victimization, community violence exposure by victimization, family violence exposure by witnessing, and community violence exposure by witnessing with regard to the variables including gender, parents’ years of schooling, types of family, family structure, place of birth, and single-child status. ANOVAs were performed to examine differences among the three age groups for the four violence exposure types. Correlation analyses were applied to investigate the correlation between violence exposure and deviant behaviors of peers. Multiple regression analysis was used to predict the main influencing factors of violence exposure among college students. The means and standard deviations (*SDs*) are reported. The standard 5% level of significance was applied for all statistical analyses.

## Results

### Characteristics of College Student Violence Exposure

The mean score of violence exposure by direct victimization at home was 3.75 (*SD* = 3.71), and 81.33% (*n* = 305) of the subjects reported that they were exposed to family violence by direct victimization at least once in their lifetime. More than four-fifths (*n* = 323, 86.13%) reported that they witnessed family violence at least once in their lifetime, with a mean score of 4.30 (*SD* = 4.27). In community violence exposure, 89.06% (*n* = 334) of college students experienced direct violence as victims at least once in their lifetime with a mean score of 5.09 (*SD* = 4.30), and almost all the participants (*n* = 374, 99.73%) were exposed to community violence by witnessing at least once in their lifetime with a mean score of 10.58 (*SD* = 6.86).

Paired sample *t*-tests revealed that subjects obtained higher scores on violence exposure both by victimization in community (*t* = −6.45, *df* = 374, *p* = 0.000, 95% confidence interval [CI] [−1.75, −0.93], Cohen’s *d* = −0.33) and witnessing in community (*t* = −18.25, *df* = 374, *p* = 0.000, 95% CI [−6.96, −18.25], Cohen’s *d* = -1.1) than they did at home.

### Comparisons of Violence Exposure With Sociodemographic Variables

The comparison results of family violence exposure and community violence exposure on college students with sociodemographic variables are presented in Tables 2, 3, respectively.

**TABLE 2:** Family violence exposure in college students: sociodemographic comparison.

**Sociodemographic variables**		**Family violence exposure by witnessing**						**Family violence exposure by victimization**					
							**Effect size**						**Effect size**
	***n* (375)**	***M* ± *SD***	***t or F***	***df***	***p*-value**	**95% CI**	**Cohen’s *d* or *f***	***M* ± *SD***	***t or F***	***df***	***p*-value**	**95% CI**	**Cohen’s *d* or *f***
Age	≤19 (133)	4.34 ± 4.71	*F* = 0.016	374	0.984	*–*	*f* = 0.01	3.80 ± 3.80	*F* = 0.027	374	0.974	*–*	*f* = 0.01
	20 (97)	4.24 ± 4.48						3.73 ± 3.74					
	≥21 (145)	4.30 ± 3.71						3.70 ± 3.63					
Gender	Male (222)	4.25 ± 4.64	*t* = −0.26	373	0.793	[-1, 0.77]	*d* = −0.03	4.06 ± 3.76	*t* = 2.0*	373	0.047	[0.01, 1.54]	*d* = 0.21
	Female (153)	4.37 ± 3.69						3.29 ± 3.60					
Family structure	Single-parent family (18)	4.83 ± 4.15	*t* = 0.55	373	0.585	[−1.47, 2.60]	*d* = 0.13	5.33 ± 5.99	*t* = 1.17	18	0.258	[−1.33, 4.66]	d = 0.34
	Two-parent family (357)	4.27 ± 4.28						3.67 ± 3.55					
Father’s years of	≤12 (230)	4.63 ± 4.28	*t* = 1.89	373	0.59	[−0.34, 1.74]	*d* = 0.20	4.09 ± 3.78	*t* = 2.25*	373	0.025	[0.11, 1.65]	*d* = 0.24
schooling	≥13 (145)	3.77 ± 4.23						3.21 ± 3.55					
Mother’s years of	≤12 (265)	4.35 ± 3.87	*t* = 0.36	373	0.719	[−0.78, 1.13]	*d* = 0.04	3.97 ± 3.70	*t* = 1.84	373	0.066	[−0.51, 1.6]	*d* = 0.21
schooling	≥13 (110)	4.17 ± 5.12						3.20 ± 3.69					
Types of family	Nuclear family	3.94 ± 4.18	*t* = −1.75	373	0.080	[−1.64, 0.10]	*d* = −0.18	3.48 ± 3.48	*t* = −1.51	350	0.133	[−1.34, 0.18]	*d* = −0.16
	Non-nuclear family	4.71 ± 4.35						4.06 ± 3.94					
Place to grow up	Urban area (78)	4.00 ± 4.83	*t* = 0–0.69	373	0.492	[−1.44, 0.70]	*d* = −0.08	2.95 ± 3.34	*t* = −2.14*	373	0.033	[−1.93,-0.84]	*d* = −0.28
	Rural area (297)	4.37 ± 4.12						3.96 ± 3.78					
Single-child status	Only child (212)	3.82 ± 3.98	*t* = −2.51*	317	0.013	[−2.03,-0.24]	*d* = −0.26	3.54 ± 3.70	*t* = −1.34	371	0.181	[−1.28, 0.24]	*d* = −0.14
	Not only child (161)	4.95 ± 4.58						4.06 ± 3.71					

Variance “age” used the analysis of variance, and the rest using independent sample T-test. 95% CI = 95% confidence interval. *p < 0.05.

**TABLE 3:** Community violence exposure in college students: sociodemographic comparison.

**Sociodemographic variables**			**Community violence exposure by witnessing**					**Community violence exposure by victimization**					
							**Effect size**						**Effect size**
	***n* (375)**	***M* ± *SD***	***t or F***	***df***	***p*-value**	**95% CI**	**Cohen’s *d* or *f***	***M* ± *SD***	***t or F***	***df***	***p*-value**	**95% CI**	**Cohen’s *d* or *f***
Age	≤19 (133)	10.17 ± 6.74	*F* = 0.427	374	0.653	*–*	*f* = 0.05	5.10 ± 4.45	*F* = 0.944	374	0.390	*–*	*f* = 0.07
	20 (97)	10.98 ± 7.94						5.55 ± 4.47					
	≥21 (145)	10.70 ± 6.18						4.77 ± 4.03					
Gender	Male (222)	12.04 ± 7.41	*t* = 5.44***	372	0.000	[2.28, 4.87]	*d* = 0.53	6.13 ± 4.76	*t* = 6.4***	369	0.000	[1.76, 3.33]	*d* = 0.65
	Female (153)	8.46 ± 5.33						3.58 ± 2.93					
Family structure	Single-parent family (18)	10.22 ± 5.28	*t* = −0.23	373	0.820	[−3.64, 2.89]	*d* = −0.06	4.33 ± 3.66	*t* = −0.76	373	0.446	[−2.83, 1.25]	*d* = −0.20
	Two-parent family (357)	10.60 ± 6.94						5.13 ± 4.33					
Father’s years of	≤12 (230)	11.18 ± 7.12	*t* = 2.15*	373	0.032	[0.13, 2.98]	*d* = 0.23	5.52 ± 4.41	*t* = 2.45*	373	0.015	[0.22, 2.0]	*d* = 0.26
schooling	≥13 (145)	9.63 ± 6.33						4.41 ± 4.03					
Mother’s years of	≤12 (265)	10.92 ± 6.77	*t* = 1.51	373	0.133	[−0.36, 2.7]	*d* = 0.17	5.32 ± 4.36	*t* = 1.63	373	0.103	[−0.16, 1.75]	*d* = 0.19
schooling	≥13 (110)	9.75 ± 7.03						4.53 ± 4.11					
Types of family	Nuclear family	10.03 ± 6.51	*t* = −1.67	373	0.096	[−2.57, 0.21]	*d* = −0.17	5.01 ± 4.42	*t* = −0.40	373	0.690	[1.05, 0.70]	*d* = −0.04
	Non-nuclear family	11.21 ± 7.21						5.18 ± 4.16					
Place to grow up	Urban area (78)	9.03 ± 6.27	*t* = −2.26*	373	0.024	[−3.67,-0.26]	*d* = −0.30	4.27 ± 4.10	*t* = −1.90	373	0.058	[−2.10, 0.037]	*d* = −0.24
	Rural area (297)	10.99 ± 6.96						5.30 ± 4.33					
Single-child status	Only child (212)	10.06 ± 6.48	*t* = −1.72	371	0.086	[−2.64, 0.18]	*d* = −0.18	5.24 ± 4.62	*t* = 0.72	368	0.473	[−0.55, 1.18]	*d* = 0.07
	Not only child (161)	11.29 ± 7.29						4.93 ± 3.85					

95% CI = 95% confidence interval. Variance “age” used the analysis of variance, and the rest using independent sample t-test. Because there is no significant difference in variance analysis among the three groups of variable age, there are no post-hoc multiple comparisons, and so no 95% confidence interval values. * p < 0.05, *** p < 0.001.

For family violence exposure by witnessing, an independent sample *t-*test showed that subjects with siblings had a significantly higher score than those who were the only child in the family (*t* = −2.51, *df* = 317, *p* = 0.013, 95% CI [−2.03, −0.24], Cohen’s *d* = −0.26).

For family violence exposure by victimization, the results of independent sample *t-*tests showed that male students scored significantly higher than female students (*t* = 2.0, *df* = 373 *p* = 0.047, 95% CI [0.01, 1.54], Cohen’s *d* = 0.21); subjects with father’s years of schooling ≤ 12 scored significantly higher than those with fathers having ≥13 years of education (*t* = 2.25, *df* = 373, *p* = 0.025, 95% CI [0.11, 1.65], Cohen’s *d* = 0.24); and college students who grew up in rural areas had significantly higher scores than those in urban areas (*t* = −2.14, *df* = 373, *p* = 0.033, 95% CI [−1.93, −0.84], Cohen’s *d* = −0.28).

For community violence exposure by witnessing, the significant statistical results were similar to those for family violence exposure by victimization. The results of independent sample *t*-test indicated that male students had higher score than female students (*t* = 5.44, *df* = 372, *p* = 0.000, 95% CI [2.28, 4.87], Cohen’s *d* = 0.53); subjects with father’s years of schooling ≤ 12 scored significantly higher than those with father having ≥13 years of education (*t* = 2.15, *df* = 373, *p* = 0.032, 95% CI [0.13, 2.98], Cohen’s *d* = 0.23); and college students who grew up in rural areas obtained significantly higher scores than those in urban areas (*t* = −2.26, *df* = 373, *p* = 0.024, 95% CI [−3.67, −0.26], Cohen’s *d* = −0.30).

For community violence exposure by victimization, independent sample *t*-tests showed that male students scored significantly higher than female students (*t* = 6.40, *df* = 369 *p* = 0.000, 95% CI [1.76, 3.33], Cohen’s *d* = 0.65) and subjects with father’s years of schooling ≤12 scored significantly higher than those with fathers having ≥13 years of education (*t* = 2.45, *df* = 373, *p* = 0.015, 95% CI [0.22, 2.0], Cohen’s *d* = 0.26).

There were no significant differences for other sociodemographic factors.

### Correlations Between Deviant Behaviors of Peers and Violence Exposure of College Students

Table 4 lists the correlation analysis results between deviant behaviors of peers and violence exposure of college students. Deviant behaviors of peers were significantly related to total family violence exposure (*r* = 0.312, *df* = 373 *p* < 0.01) and family violence exposure by witnessing (*r* = 0.229, *df* = 373 *p* < 0.01) and by victimization (*r* = 0.292, *df* = 373, *p* < 0.01).

**TABLE 4:** Correlations between peers’ deviant behaviors and college students’ violence exposure (*r*, *n* = 375).

	***M ± SD***	**Peers’ deviant behaviors**
Domestic violence exposure by witnessing	4.304.27	0.229**
Domestic violence exposure by victimization	3.753.71	0.292**
Total domestic violence exposure	8.046.60	0.312**
Community violence exposure by witnessing	10.586.86	0.472**
Community violence exposure by victimization	5.094.30	0.406**
Total community violence exposure	15.679.97	0.500**
Deviant behaviors of peers	0.550.32	

** *p* < 0.01.

Deviant behaviors of peers were also significantly associated with total community violence exposure (*r* = 0.500, *df* = 373, *p* < 0.05) and community violence exposure by witnessing (*r* = 0.472, *df* = 373, *p* < 0.01) and by victimization (*r* = 0.406, *df* = 373, *p* < 0.01).

### Multiple Regression Analysis of Factors Related to Violence Exposure

The dependent variable was total score on the violence exposure. There were 10 independent variables including deviant behaviors of peers and 9 sociodemographic variables, such as age, gender, family structure, types of family, location of growing up, single-child status, length of stay with family members, and years of parental education. Five were binary variables: gender (male = 1, female = 0), family structure (single-parent family = 1, two-parent family = 0), types of family (nuclear family = 1, non-nuclear family = 0), location of growing up (urban = 1, rural = 0), single-child status (only child = 1, siblings = 0). Stepwise multiple regression was performed, and the results are shown in Table 5. The top three factors related to violence exposure were deviant behaviors of peers, gender, and single-child status, explaining 29.1% of the variance. The remaining seven variables (age, family structure, types of family, places of growing up, length of stay with family members, the length of years that their parents receive education) were not significant (*p* < 0.05).

**TABLE 5:** Multiple regression analysis of the factors related to violence exposure in college students.

**Variable**	**Unstandardized**		**Standardized**	***t***	***P***	**95% confidence interval**	
	***B***	***SE***	**BETA**			**Lower bound**	**Upper bound**
Constant	29.006	11.561		2.509	0.013	6.271	51.742
Gender	3.298	1.333	0.115	2.475	0.014	0.678	5.918
Age	–0.713	0.476	–0.071	–1.497	0.135	–1.650	0.223
Family structure	–3.045	2.968	–0.046	–1.026	0.306	–8.881	2.791
Father’s years of schooling	–0.888	0.991	–0.059	–0.896	0.371	–2.837	1.061
Mother’s years of schooling	0.177	1.158	0.011	0.153	0.878	–2.100	2.455
Length of stay with family	–0.066	0.247	–0.014	–0.269	0.788	–0.551	0.419
Family type	–1.384	0.822	–0.102	–1.685	0.093	–3.000	0.231
Place of growing up	0.953	1.139	0.051	0.837	0.403	–1.287	3.193
Single-child status	4.426	1.864	0.158	2.375	0.018	0.761	8.091
Deviant behaviors of peers	21.121	2.012	0.484	10.498	0.000	17.164	25.077

**Adjusted R^2^ = 0.291.**

## Discussion

Exposure to violence is considered a pervasive public problem among children and adolescents (Stein et al., 2003; Gellman and Delucia-Waack, 2006; Brady et al., 2008; Finkelhor et al., 2009). However, there is a lack of research focusing on violence exposure in late adolescents and young adults, including in China. This research addressed this gap, evaluating both family and community violence exposure by victimization and witnessing among college students. In America, prevalence rates of violent victimization during adolescence are estimated to be 50–68% (Menard, 2002; Macmillan and Hagan, 2004). In Finkelhor et al.’s study (2009), 41.2% of children and youth reported they had experienced direct physical assault in their lifetime. The current research estimated the prevalence of violence exposure more concretely and found that more than four-fifths of the participants were exposed to family violence and almost 9 in 10 were exposed to community violence as direct victims at least once in their lifetime, values that were slightly higher than in western countries. This finding indicated that family and community violence exposure of college students cannot be ignored given its high occurrence rate and serious adverse consequences.

One group reported that ∼90% of youth were exposed to violence in school (Flannery et al., 2004) and another stated that almost 80% have been exposed to violence in community (Weist et al., 2001). Although violence exposure in families is more difficult to estimate, rates of 17–25% have been reported (O’Brien et al., 1994; Hotton, 2003). In this research, the percentages of adolescents exposed to community violence by witnessing and victimization were quite similar to the studies above (Weist et al., 2001), but the rate of family violence exposure was much higher than existing studies (O’Brien et al., 1994; Hotton, 2003). We also found that participants obtained higher scores on community violence exposure than they did in family, both by victimization and witnessing.

Our results show that individuals with different sociodemographic backgrounds had different scores in family and community violence exposure by victimization and witnessing. Firstly, male students were exposed more to family and community violence than female students were. This is consistent with previous study results describing that males experienced significantly more witnessed violence and physical assaults than did females in their lifetime (Coie and Dodge, 1997; Xie et al., 2011). Second, college students with less-educated fathers had higher scores as victims of family violence exposure and as victims and witnesses of community violence exposure than those whose fathers had more education. The results of some studies also found that the parents of young violent offenders had lower education levels and graduated from elementary or junior high school (Huesmann et al., 2002; Song et al., 2003; Chang et al., 2014). Thirdly, college students from urban areas had lower exposure rates to family and community violence than those from rural areas. The families of adolescents with violent behavior usually had poor economic conditions and lived in rural areas since childhood (Chang et al., 2014). Fourthly, subjects with siblings were more likely to be exposed to family violence than were those who were the only child in the family. Conflicts often occurred in families with many children, and their parents usually ridiculed, scolded, and imposed corporal punishment to solve the problems (Chan, 1994; Song et al., 2003; Gibson, 2012; Yoo and Huang, 2012).

The majority of domestic violence victims were female, while perpetrators were usually male. Men who committed violence against women were often less educated (Envuladu et al., 2012; Kunnuji, 2014). Another study concluded that having more children can increase the risk of child abuse by the parent (Chan, 1994; Gibson, 2012; Yoo and Huang, 2012). However, scientific consensus on this issue has not been reached. The studies of Widom (2000) and Zhao et al. (2008) found that children who were abused and neglected during their childhood did not commit violent crimes or conduct bad behavior during their adulthood, which was inconsistent with the results of our study. Further research should be conducted in this area to clarify these discrepancies.

Apart from family environment, the three variables of father’s years of schooling, location of growing up, and single-child status are closely interrelated in our results. This is consistent with Chang et al.’s study results (2014). A male with less education is more likely to live in rural areas and have more than one child due to the influence of Chinese Family Planning policy (Chang et al., 2014). In rural areas of China, educational resources are usually scarce compared with urban cities (Gan and Huang, 2006). Those who receive less education are highly likely to form their family in rural areas and maintain the vicious circle for generations (Gan and Huang, 2006). They would have little knowledge of modern education theory, and their concepts and parenting styles are often unscientific and crude (Chang et al., 2014). Especially when there is more than one child in a family and parents need to divide their time and attention, they may neglect a certain child or to treat them rudely (Chan, 1994; Song et al., 2003; Gibson, 2012; Yoo and Huang, 2012). Therefore, improving parental style and creating a warm family atmosphere could be essential points to reduce family violence exposure and prevent violent behaviors in adolescents (Bu et al., 2017; Lan et al., 2019).

Being in the “weaning period,” adolescents are attempting to be independent and prefer to spending more time with their friends than with their family. Peer groups therefore have profound effects on the socialization process of this age group (Ryan, 2000; Iervolino et al., 2002). We found that deviant behaviors of peers were significantly associated with exposure to family and community violence by both witnessing and victimization. More peer deviant behaviors were associated with higher levels of violence. As the main form of adolescents’ social interaction, deviant behaviors by peers can deeply impact the violence exposure of adolescents, which is linked to multiple levels of behavioral and psychosocial development (Schwartz et al., 2003; Mrug et al., 2008).

Multiple regression analysis indicated that deviant behaviors of peers and sociodemographic variables were the primary factors associated with college student violence exposure. We found that male respondents were more likely to be exposed to violence, and those who have peers with more deviant behaviors and are not the only child in their families had higher violence exposure.

Spending most of their time in schools, adolescents are easily influenced by their peers, and it is difficult to stop them from spending time with their peers after they become friends. The theory of social connection (Hirschi, 1969) posits that parental supervision of adolescents can significantly influence the interpersonal relationships of their children. Those supervised by parents would be less likely to have friends with problematic behaviors, thus reducing the possibility of bad behavior in their own life. Considering the characteristics of students of different ages, the school would regularly provide distinguishing training and guidance on parent supervision methods, such as holding lectures or workshops, to help parents enrich supervision knowledge and learn from each other through discussions. At the same time, schools may provide courses to train young people to resolve conflicts when they face violence.

Generally speaking, adolescent boys are often more impulsive and like to experience adventure, making it easier for them to engage in violence than girls (Zhang et al., 2004). Other studies indicated that there is more tolerance of male violence in the social culture (Yang and Ye, 2006). To improve the situation, parents and teachers should pay special attention and give timely guidance to young boys to encourage them to express and vent negative emotions in an appropriate way, such as doing exercise and improving self-control (Zhang et al., 2004; Li, 2016).

Additionally, universities should play a role in preventing violence. University security departments may regularly provide training sessions for faculty and students to help them identify and respond to violence properly and reduce the spread of violence (O’Neill, 2008; Yavuzer and Gundogdu, 2012). Timely and professional psychological counseling should also be easily accessible to those who have emotional problems and help them to deal with impulsive violent outbursts. It is essential to build a healthy, harmonious, and safe campus environment for students, and all parties should take relevant actions.

This research is subject to several limitations. The first is that the sample was only selected from college students in the Shaanxi Province, so the results may not be generalized nationwide. The second concerns the retrospective method used to collect data. The results may therefore be affected by recall bias. The third limitation is the small sample size due to time and resource constraints. In future research, we need to increase the sample size and adopt more methods to study this issue.

In addition to the above-listed variables, the effects of other social environment factors should also be considered, such as media violence information on the internet. As the use of internet spreads widely and plays an increasingly important role in daily life, future research should assess violence exposure on the Internet to obtain a more comprehensive understanding of violence exposure and its influence on adolescents.

Given the high level of violence exposure among Chinese college students, more in-depth investigations into victimization and witnessing should be conducted across multiple contexts (in the family, community, and on the internet) for adolescents in China or even around the world. This will lead to more effective methods of preventing violence exposure.

## Conclusion

Due to the high levels of family and community violence exposure among Chinese college students by both victimization and witnessing, researchers, doctors, and government officials should take their responsibilities and make more effort to improve the situation. Family environments (including child abuse and intimate partner violence) and social environments (especially deviant behaviors of peers) are closely related to real-life violence exposure to adolescents. Parents and teachers should provide timely and appropriate guides to avoid possible violence exposure and propose suggestions to cope with the existing violence exposure in teenagers’ daily lives. More patience and understanding should also be shown to adolescents in a stage with changing cognition and emotion to protect their mental and psychological health.

## Data Availability Statement

The raw data supporting the conclusions of this article will be made available by the authors, without undue reservation, to any qualified researcher.

## Ethics Statement

The studies involving human participants were reviewed and approved by Biomedical Ethics Committee of Medical Department of Xi’an Jiaotong University. Written informed consent to participate in this study was provided by the participants’ legal guardian/next of kin. Written informed consent was obtained from the individual(s), and minor(s)’ legal guardian/next of kin, for the publication of any potentially identifiable images or data included in this article.

## Author Contributions

WW participated in the design of the study and drafted the manuscript. YW also participated in the design of the study. YQ performed the statistical analysis. YY was involved in the data collection. All authors approved the final manuscript.

## Conflict of Interest

The authors declare that the research was conducted in the absence of any commercial or financial relationships that could be construed as a potential conflict of interest.
